# A comprehensive look at the COVID-19 pandemic death toll

**DOI:** 10.7554/eLife.71974

**Published:** 2021-08-12

**Authors:** Lone Simonsen, Cecile Viboud

**Affiliations:** 1 PandemiX Center, Institute of Science and Environment, Roskilde University Roskilde Denmark; 2 Fogarty International Center, National Institutes of Health Bethesda United States

**Keywords:** COVID, mortality, data, international, Human

## Abstract

COVID-19 ‘excess mortality’ has been estimated for more than 100 countries and shows a dramatic death toll in many countries.

**Related research article** Karlinsky A, Kobak D. 2021. Tracking excess mortality across countries during the COVID-19 pandemic with the world mortality dataset. *eLife* **10**:e69336. doi: 10.7554/eLife.69336

More than 18 months into the pandemic, the exact death toll of COVID-19 remains elusive. There are several ways to assess how many people have died in a pandemic, each with their advantages and disadvantages. Official national reports of COVID-19 deaths are useful, but their accuracy depends on the level of testing in that country and may underestimate the true death toll. Combining mortality rates with estimates of the fraction of people in a country who have been infected provides a better estimate, but requires serological studies of antibody prevalence that are not widely available. A tried-and-trusted approach to calculate the death toll is to estimate 'excess mortality' by comparing the total number of deaths during the pandemic period with a baseline level of deaths before the pandemic. This indirect statistical approach does not depend on testing strategy.

A recent study, based on data from 29 high-income countries during 2020, reported substantial excess mortality in some Eastern European countries and no excess mortality in New Zealand, Norway or Denmark (Islam et al., 2021). Now, in eLife, Ariel Karlinsky (Hebrew University) and Dmitry Kobak (University of Tübingen) report the results of an excess mortality study that extends to the summer of 2021 and more than 100 countries, and provides a first look at the substantial pandemic death toll in several middle-income countries (Karlinsky and Kobak, 2021). Excess mortality depends on infection rates, population demographics, COVID-19 interventions and vaccine coverage, and reflects the unique pandemic experience of different countries.

Karlinsky and Kobak compiled a unique database of weekly, monthly or quarterly deaths in 103 countries, including five years of pre-pandemic baselines for most of these. In some of the worst-affected countries, mortality in 2020 and the first half of 2021 exceeded the baseline level by between 50% and 150% (e.g., Peru and Mexico), and in absolute terms, more than 0.4% of the population died of COVID-19 in some countries (e.g., Peru and Bulgaria). For Peru, the most severely affected country, the effects of a poor healthcare system may have been exacerbated by strict lockdowns that fostered severe economic restraints and mass migration (Taylor, 2021). Meanwhile, countries like Japan, Finland, the Philippines, and South Korea had negative excess mortality, reflecting excellent pandemic control, which resulted in a modest COVID-19 death toll and a near absence of influenza deaths during the pandemic. For such countries, the official COVID-19 death count is more accurate than excess mortality estimates. The impact of COVID-19 was intermediate in South Africa and Russia, with mortality about 30% higher than the baseline and a death rate of about 0.3%.

Excess mortality reflects the sum of positive and negative changes from baseline years, meaning that some of the changes observed may not be directly related to the COVID-19 pandemic itself, but due instead to interventions. For example, social distancing measures decrease circulation and mortality due to influenza and other non-SARS-CoV-2 pathogens, but other factors – such as overwhelmed healthcare systems, violence and drug overdoses – increase mortality. However, Karlinsky and Kobak convincingly argue that most excess deaths reflect the direct consequences of COVID-19 (see also the study on excess death in Russia: Kobak, 2021).

Karlinsky and Kobak refrain from calculating the global mortality burden of COVID-19. Such an estimate would be heavily biased due to the lack of data for populous countries like China and India, and because many low-income countries in Asia and Africa cannot participate due to a lack of timely national vital statistics. Such gaps in the data can impact a global mortality estimate greatly: for example, one recent study computed a likely toll of about 4 million COVID-19 deaths in India, which is about 10 times higher than official death counts (Anand et al., 2021). Karlinsky and Kobak estimate that for the 103 countries they collected data for, the true number of COVID-19 deaths is on average 1.4 fold greater (range between 1 to 100-fold) than reported.

Global mortality estimates for past influenza pandemics range from 0.4 million deaths for the 2009 pandemic to 75 million deaths for the 1918 pandemic (Murray et al., 2006; Simonsen et al., 2013; Viboud et al., 2016; all adjusted to 2020 population, see Table 1). Globally, 4.3 million deaths due to SARS-CoV-2 have been reported as of August 11, 2021: this corresponds to about 6 million deaths when applying the mean underreporting factor of 1.4 reported by Karlinsky and Kobak. This is clearly a low global estimate due to missing data from key countries and the continued circulation of new SARS-CoV-2 variants in 2021 and in the coming years. Even so, the COVID-19 pandemic is already deadlier than the 1957 pandemic, but has nowhere near the death toll of the pandemic of 1918.

**Table 1.** Estimates of global excess mortality for five pandemics. Estimates of the global per capita excess mortality rate (row 2), the number of global excess deaths adjusted to 2020 population (row 3), and the mean age at death (row 4) for the ongoing COVID-19 pandemic (column 2) and the influenza pandemics of 2009, 1968, 1957 and 1918 (columns 3–6). Each study used different statistical models, assumptions and country data. The levels of mortality in non-participating countries were estimated using various extrapolation/imputation strategies.

	COVID-19^1^	2009^2^	1968^3^	1957^4^	1918^5^
Per capita excess mortality rate	>0.08%^6^	0.005%	0.03%	0.04%	1.0%
Global excess deaths adjusted to 2020 population	>6 million^6^	0.4 million	2.2 million	3.1 million	75 million
Mean age at death (years)	70^7^	37	62	65	27

^1^Karlinsky and Kobak, 2021. 103 wealthier countries; an under-reporting factor of 1.4 was applied. ^2^Simonsen et al., 2013. Based on 2009 data from 20 countries covering approximately 35% of the world population and using an allcause imputation method that uses 10 factors. Estimates based on 300,000–400,000 pandemic excess deaths from all causes.

^3^CDC, 2019. Based on 1 million excess deaths in the US, UK, Canada, Australia and France. ^4^Viboud et al., 2016. For the entire pandemic period (1957–1959), using data from 39 countries; extrapolated globally based on GDP, latitude and baseline death rate.

^5^Murray et al., 2006. 13 countries or regions for the entire pandemic period between 1918–1920; allcause mortality; extrapolated by GDP, latitude; (62 million deaths in 2004 population). ^6 ^Based on the official COVID-19 global death toll as of 11/8/2021 multiplied by 1.4 to allow account for underreporting (Karlinsky and Kobak, 2021). This is an underestimate as the 103 participating countries in this study are wealthier, but harder-hit populous countries like India (which may account for approximately 4 million excess deaths) are not included. Also, the burden is incomplete because the COVID-19 pandemic is not yet over.

^7^The COVID-19 pandemic mainly kills the elderly, but the exact mean age of deaths is not currently known. Mean age at death is likely lower in middle-income countries: for example, it is reported to be 60 years in South Africa (Guimarães et al., 2021; Statistics South Africa, 2020).

Importantly, these historical comparisons do not consider long-term decreases in baseline mortality due to better healthcare, longer life expectancy and other factors, which make the COVID-19 pandemic stand out sharply against low background mortality levels. Another key consideration is age: the mean age of people who die of COVID-19 is around 70 years, similar to the 1957 pandemic, but dramatically higher than the 1918 and 2009 pandemics (Table 1). The mean age at death is likely lower for less wealthy countries with younger populations (e.g., 60 years in South Africa; Table 1). Mortality age patterns are critically needed for estimating years of life lost, which is an alternative metric used to understand and compare pandemic death tolls (Viboud et al., 2010; Pifarré I Arolas et al., 2021).

We applaud Karlinsky and Kobak’s efforts to compile, release and update timely mortality data in over 100 countries – a major achievement that would have been impossible even 10 years ago. This is a data revolution that parallels that seen in vaccine development and pathogen sequencing. Future work should focus on including incomplete or subnational mortality data from low- and middle-income countries in Asia, the Middle East and Africa (United Nations) to begin filling the data gap. The database should also be expanded to include age breakdowns whenever available, as in other international mortality databases (such as the Human Mortality Database, COVerAGE-DB, and EuroMOMO). Going forward, we call for resources to maintain these valuable databases in the post-SARS-CoV-2 era, as these can uniquely monitor the complete impact of the COVID-19 pandemic. These databases can also be used to track the death toll of increasingly frequent heat waves and other effects of climate change, and help us be ready for future pandemics.

## Note

Disclaimer: This article does not necessarily represent the views of the NIH or the US government.

## References

[bib1] Anand A, Sandefur J, Subramanian A (2021). Three new estimates of India’s all-cause excess mortality during the COVID-19 pandemic. Center for Global Development.

[bib2] CDC (2019). 1968 pandemic (H3N2 virus). https://www.cdc.gov/flu/pandemic-resources/1968-pandemic.

[bib3] Guimarães RM, Portela MC, Villela DAM, Correa Matta G, de Freitas CM (2021). Younger Brazilians hit by COVID-19 – What are the implications?. The Lancet Regional Health - Americas.

[bib4] Islam N, Shkolnikov VM, Acosta RJ, Klimkin I, Kawachi I, Irizarry RA, Alicandro G, Khunti K, Yates T, Jdanov DA, White M, Lewington S, Lacey B (2021). Excess deaths associated with covid-19 pandemic in 2020: age and sex disaggregated time series analysis in 29 high income countries. BMJ.

[bib5] Karlinsky A, Kobak D (2021). Tracking excess mortality across countries during the COVID-19 pandemic with the world mortality dataset. eLife.

[bib6] Kobak D (2021). Excess mortality reveals Covid's true toll in Russia. Significance.

[bib7] Murray CJL, Lopez AD, Chin B, Feehan D, Hill KH (2006). Estimation of potential global pandemic influenza mortality on the basis of vital registry data from the 1918–20 pandemic: a quantitative analysis. The Lancet.

[bib8] Pifarré I Arolas H, Acosta E, López-Casasnovas G, Lo A, Nicodemo C, Riffe T, Myrskylä M (2021). Years of life lost to COVID-19 in 81 countries. Scientific Reports.

[bib9] Simonsen L, Spreeuwenberg P, Lustig R, Taylor RJ, Fleming DM, Kroneman M, Van Kerkhove MD, Mounts AW, Paget WJ, GLaMOR Collaborating Teams (2013). Global mortality estimates for the 2009 influenza pandemic from the GLaMOR project: a modeling study. PLOS Medicine.

[bib10] Statistics South Africa (2020). COVID-19 pandemic in South Africa. http://www.statssa.gov.za/publications/Report%2000-80-05/Report%2000-80-052020.pdf.

[bib11] Taylor L (2021). Covid-19: why Peru suffers from one of the highest excess death rates in the world. BMJ.

[bib12] Viboud C, Miller M, Olson DR, Osterholm M, Simonsen L (2010). Preliminary estimates of mortality and years of life lost associated with the 2009 A/H1N1 pandemic in the US and comparison with past influenza seasons. PLOS Currents.

[bib13] Viboud C, Simonsen L, Fuentes R, Flores J, Miller MA, Chowell G (2016). Global mortality impact of the 1957–1959 influenza pandemic. Journal of Infectious Diseases.

